# Usability and Usefulness of Occupational Health Care Patient Portals: Patient-Based Cross-Sectional Study

**DOI:** 10.2196/73197

**Published:** 2025-08-14

**Authors:** Sari Nissinen, Pauliina Toivio, Erja Sormunen

**Affiliations:** 1 Finnish Institute of Occupational Health, Topeliuksenkatu 41b, Helsinki, 00250, Finland, 358 438252435

**Keywords:** patient portal, patient care, occupational health, work ability data, online questionnaire, Finland, descriptive statistics, general linear regression

## Abstract

**Background:**

Patient portals are a crucial part of modern health care services, as they provide patients with access to their personal information and enable communication with health care professionals. The usability and usefulness of these portals are decisive factors in their adoption. There is a lack of previous research on the use of patient portals in occupational health care (OHC).

**Objective:**

This study aims to examine patients’ experiences with the usability and usefulness of OHC patient portals and to identify the factors that influence their perceived usability and usefulness.

**Methods:**

A cross-sectional study was conducted through a web-based survey in April 2024 in Finland. Of the 3072 respondents, usability was assessed using 12 statements, and usefulness was evaluated with 9 statements. Responses were collected on a 5-point Likert scale. The survey also gathered respondents’ background information, including age, gender, education, information and communication technology (ICT) skills, satisfaction, frequency of use, and data privacy concerns. Data analysis was performed using SPSS Statistics (version 29; IBM Corp), applying frequency analysis and a general linear model.

**Results:**

The results showed that 75.1% (1895/2523) of respondents agreed that the portal was easy to learn and use, 70.5% (1774/2517) felt it supported collaboration with OHC, while 52.4% (1316/2511) reported that it provided a good overview of their work ability, and 35.5% (891/2505) felt it offered a good overview of their working conditions. The perceived usability of OHC patient portals was significantly associated with several factors in the adjusted model: fear of unauthorized access to data (*F*_4_=4.49, *P*=.001, η²=0.007), need for guidance (*F*_4_=52.2, *P*<.001, η²=0.080), frequency of use (*F*_1_=34.3, *P*<.001, η²=0.014), satisfaction with the portal (*F*_1_=577.1, *P*<.001, η²=0.193), perceived ICT skills (*F*_1_=12.2, *P*<.001, η²=0.005), and age (*F*_1_=10.8, *P*<.001, η²=0.004), with younger users (≤50 years) reporting better usability. Perceived usefulness was significantly influenced by frequency of use (*F*_1_=9.80, *P*=.002, η²=0.004) and satisfaction (*F*_1_=548.0, *P*<.001, η²=0.183), while other factors such as fear of unauthorized access (*F*_4_=0.41, *P*=.80), need for guidance (*F*_4_=1.52, *P*=.20), ICT skills, education, gender, and age were not statistically significant.

**Conclusions:**

OHC patient portals must enhance their capacity to provide information on work ability and working conditions. Improved documentation of work-related hazards, risks, and workload factors in medical records is needed. These enhancements could raise patient awareness of work well-being factors.

## Introduction

Generally, a patient portal is a website or mobile app that provides patients with access to their personal health information using a username and password. Through the portal, patients can view health records documented by health care professionals, such as prescribed medications, administered vaccinations, identified allergies, and laboratory test results. Additionally, the portal might enable secure communication with health care professionals [1-3].

Usability and usefulness are key factors in the adoption of patient portals [4-7]. Positive experiences are often associated with the portal’s usability and useful features, such as the ability to request prescription renewals and view visit documentation [2,6,8]. Real-time access to information is also an essential aspect of a good user experience [5,6]. Some patients find portals easy to use and useful, while others encounter challenges due to complex interfaces [5,9], lack of user training [10], doubts about the portal’s usefulness [11,12], and comprehensibility of medical terminology [8]. Concerns about security and privacy [2,5,12,13] or lack of professional recommendations may also reduce willingness to use portals [1,14].

The impact of patient portal use on health outcomes has been variable. Some studies [15-17] have found no significant effect of portal use on health outcomes. Conversely, some studies have reported improvements in areas such as quality of care [18], health behavior [19], and understanding own health [5,20]. Many other studies indicate that patient portals enhance communication between patients and health care professionals and increase patient engagement in their own care [5,8,21]. Furthermore, patient portals have helped patients identify errors in their health records, enhancing patient safety [5,22,23].

My Kanta is the national patient portal accessible to all citizens in Finland, and citizens are widely using My Kanta, which allows them to view their medical records and prescriptions, as well as check laboratory and radiology results [24,25]. Alongside My Kanta, occupational health care (OHC) providers in Finland have developed patient portals for their clients [26], likely to strengthen customer relationships by offering their own portals [27].

In Finland, legislation mandates that all employers provide preventive OHC services to their employees to prevent work-related health issues and promote employees’ health, safety, and work ability. In addition to the mandatory preventive services, employers also have the option to offer primary health care services at the general practitioner level. Thus, a significant user group for OHC portals consists of working-age patients, as around 91% of Finnish employees are covered by OHC services [28].

Similar to My Kanta, OHC patient portals are used to view medical records, prescriptions, vaccinations, and to check laboratory and radiology results. However, OHC portals, available as both a mobile app and a web service, expand on these functions by also allowing appointment booking, viewing sick leave certificates, and the designated OHC team, as well as enabling remote consultations and secure messaging [6,27].

Information in the portal is based on clinical data entered by OHC professionals and automatically transferred from the electronic health record system. This data is presented as read-only to patients. Additionally, patients are able to input their own health questionnaire responses, self-measurements, and treatment records from other health care providers. However, this patient-generated data is not visible to professionals without the patient’s explicit consent. Access rights are clearly defined. Clinical data in the portal can be updated by OHC professionals, while patients are only able to manage their own entries. Furthermore, the portal does not display records from other health care providers, such as My Kanta [25].

A previous study has shown that in OHC, a significant amount of work ability data is generated through patient work [29], as a central objective of OHC is to prevent occupational illnesses and accidents, as well as to maintain and promote work ability [30].

Despite the significant role of patient portals in health care, there is a notable gap in research regarding the experiences of OHC patients with these portals. Only 1 study has explored the experiences of patients from a single OHC provider; however, the patients’ ages ranged between 56 and 70 years, and the focus was on using patient portals for health promotion [3]. According to the study, patients were willing to use portals if the information is readily accessible and the functionalities are seamless.

Therefore, this study aims to examine the experiences of OHC patients concerning the usability and usefulness of patient portals. We are also interested in identifying the factors that influence the usability and perceived usefulness of these portals, thereby addressing the existing research gap.

The research questions (RQs) are:

RQ1: How do patients perceive the usability and usefulness of OHC patient portals?

RQ2: Which patient factors are associated with the usability and usefulness of OHC patient portals?

## Methods

### Participants and Data Collection

The study was conducted through an online survey in April 2024. Two organizations participated in the study: a trade union and a patient expert organization, both representing OHC patients. Each organization appointed a contact person who was responsible for distributing the survey. This contact person sent the survey link via email invitation to all members of their organization, with 2 reminders sent to encourage responses. Before the data collection phase, 5 individuals with experience using OHC patient portals reviewed the survey content for readability and comprehensibility. Their feedback was used to prepare the final version of the survey.

The email invitation to the potential respondents explained that this study aimed to gather insights into users’ experiences with digital patient portals in OHC to help develop customer-oriented digital services. It also noted that the survey was part of a project by the Finnish Institute of Occupational Health, supported by the Finnish Work Environment Fund. Participation was voluntary, and the estimated survey completion time was provided. The invitation emphasized respondent anonymity, assuring that personal data and organizational affiliations would not be disclosed. It also mentioned that the results would be published in a scientific paper. Additionally, the invitation included contact information, a research information sheet, and a link to the project website.

A total of 3100 individuals completed the survey. Although the exact response rate could not be determined due to the lack of precise information on how many individuals received the survey (we estimated the size of this study’s population to comprise approximately 46,000 individuals), it is known that 8372 people opened the survey link. Based on those who opened the link, the response rate can be estimated at approximately 37% (3100/8372).

### Ethical Considerations

The Ethics Committee of the Finnish Institute of Occupational Health approved this study on March 1, 2024 (approval number ETR 022024, ID154008), and the study followed the Finnish research ethics practice. All procedures followed were per the ethical standards of the responsible committee on human experimentation (institutional and national) and with the Helsinki Declaration of 1975, as revised in 2000.

Participants were not asked to provide written consent for this study. In Finland, completing the survey is generally seen as an indication of consent to participate in the research and to permit the use of the data collected. Every participant received an official cover letter detailing this study’s background, emphasizing that participation is entirely voluntary and anonymous, and noting that answering the questionnaire is considered informed consent to join this study. Also, the cover letter included the purpose of this study, the estimated time required to complete the survey, who the researchers are, the data collection methods, and the duration of data storage. The survey included a request for consent to use the responses for scientific research purposes. All responses were collected anonymously, and no identifying information was gathered. Data were stored securely as per institutional data protection policies to ensure participant privacy and confidentiality. Participants did not receive any compensation for their participation.

### Measurements

#### Dependent Variables

The perceived usability of OHC patient portals was assessed using 12 statements developed per attributes of usability: learnability, efficiency, memorability, errors, and satisfaction [31]. The perceived usefulness of OHC patient portals was evaluated with 9 statements, which were based on researchers’ experiences with OHC services and also used elements from the Technology Acceptance Model [32]. Responses to these statements were collected using a 5-point Likert scale, categorized as follows: “totally disagree,” “somewhat disagree,” “neither agree nor disagree,” “somewhat agree,” and “totally agree.”

All survey variables that investigated the usability (12 statements) and usefulness (9 statements) of OHC patient portals were combined into 2 separate sum variables. Before forming these sum variables, the internal consistency of the items was assessed using the Cronbach α coefficient, resulting in values of 0.95 for the usability sum variable and 0.93 for the usefulness sum variable.

#### Independent Variables

The survey used in this study included questions on the respondents’ background information: gender, age, education, information and communication technology (ICT) skills, satisfaction with OHC patient portals, frequency of use, a need for guidance, and fear of unauthorized persons seeing the data. These were used only in statistical analysis as adjustments.

For analyses, the variables were recoded to facilitate interpretation and enhance clarity. Gender was originally categorized as 1=female, 2=male, and 3=other, or do not want to say. For analysis purposes, these categories were recoded into 1=women and 2‐3=other. Age was initially divided into 5 groups: 1=up to 30 years, 2=31‐40 years, 3=41‐50 years, 4=51‐60 years, and 5=61 years or older. These were recoded into 2 categories: 1‐3=50 years or younger and 4‐5=51 years or older. Education was originally classified with response options of 1=basic education degree (primary school), 2=matriculation examination, 3=vocational college, 4=University or University of Applied Sciences, and 5=other. For analysis, these categories were retained except the “other” category, which was excluded. Perceived ICT skills were initially rated as 1=weak, 2=moderate, and 3=good and were recoded into 1=weak and 2‐3=at least moderate for analysis. The satisfaction variable was originally measured using response options: 1=totally unsatisfied, 2=somewhat unsatisfied, 3=neither agree nor disagree, 4=somewhat satisfied, and 5=totally satisfied. These were recoded into 2 categories for analysis: 1‐3=unsatisfied and 4‐5=satisfied. Lastly, variables, need for guidance, and fear of unauthorized persons seeing the data were retained as per the originals. Additionally, the response option “neither agree nor disagree” is referred to as “neutral.”

### Data Analysis 

The survey data were analyzed descriptively using frequency analysis in SPSS Statistics (version 29; IBM Corp) software. This study’s respondent group consisted of a total of 3100 participants who answered at least 1 survey question. Of these, 639 reported regular use of the OHC patient portal, 1906 used it randomly, and 539 had never used it. Additionally, 16 did not respond to this question. The respondents’ characteristic variables were described by the absolute (n) and relative (%) frequencies. The number of respondents varies depending on the background variable. No imputation was performed for missing data.

Before analyzing this study’s data, participants who reported using the OHC patient portal were included. Respondents’ (2545/3084) attitude statements regarding the perceived usability and perceived usefulness of OHC patient portals were described by the relative (%) frequencies (RQ1). However, the number of respondents varies because not all participants answered all attitude statements regarding usability and usefulness. Missing data was not imputed.

A general linear model (GLM) was used to examine the adjusted associations of respondent characteristics with the perceived usability and perceived usefulness of OHC patient portals (RQ2). In the GLM, only those respondents who reported using the patient portal were included in the analysis (2545/3084). The number of respondents varies because only those who both used the portal and responded to the relevant question were analyzed. Additionally, the differences between the crude and adjusted models arise because, in the adjusted model, only respondents who provided complete responses to all variables included in the model were analyzed. No imputation was performed for missing data. Descriptive statistics are presented alongside GLM results to aid interpretation of effect directions and group-level differences.

In the crude models, dichotomous variables were not treated as continuous to facilitate easier interpretation and comparison of differences. Using dichotomous variables in their original form allows for more straightforward analysis and clearer understanding of the results. In the adjusted model, dichotomous variables were treated as continuous covariates to enhance the model’s stability. A model with numerous categorical variables could become unstable, leading to less reliable results.

Model selection was guided by a combination of theoretical relevance, model fit indices (eg, Akaike Information Criterion), and the principle of parsimony. Variables that did not improve model performance or interpretability were excluded, even if they were statistically significant in univariate analyses.

First, separate analyses (crude model) were conducted to examine the association of each independent variable with the dependent variable. Second, a fully adjusted multivariable model (adjusted model) was performed to measure the joint effects of all the statistically significant independent variables as covariates in the multivariable model. Collinearity diagnostics were performed, and variance inflation factor values were checked to ensure no significant multicollinearity was present among the independent variables. We chose a statistical significance level of <0.001 to apply a stricter criterion for assessing the significance of the results and to reduce the risk of false-positive results because of the large sample size. This helps to obtain more reliable and practically significant results.

## Results

### Characteristics of Respondents

Regular use of the OHC patient portal was reported by 20.7% (634/3060) of all respondents. A total of 23.3% (102/438) of men and 20.3% (528/2606) of women reported regular use. Among respondents aged 30 years or younger, 23.4% (22/94) reported regular use. Of those who were totally satisfied with the portal, 39.7% (95/239) reported regular use. Respondents with good ICT skills included 23.2% (264/1140) regular users. Among those who totally disagreed with concerns about unauthorized access, 31.2% (170/545) reported regular use. Additionally, 31.9% (273/855) respondents who did not feel a need for additional guidance reported regular use. Respondents’ background characteristics are presented in Table 1.

**Table 1.** Respondents’ characteristics.

Characteristics	Use of the OHC^a^ portal by respondents			
	Regularly, n (%)	Occasionally, n (%)	Never, n (%)	Overall
Gender	634 (20.7)	1893 (61.9)	533 (17.4)	3060 (100)
Women	528 (20.3)	1635 (62.7)	443 (17.0)	2606 (100)
Men	102 (23.3)	249 (56.8)	87 (19.9)	438 (100)
Other, or do not want to say	4 (25)	9 (56.3)	3 (18.8)	16 (100)
Age (years)	633 (20.7)	1894 (61.9)	535 (17.5)	3062 (100)
Up to 30	22 (23.4)	49 (52.1)	23 (24.5)	94 (100)
31‐40	62 (20)	202 (65.2)	46 (14.8)	310 (100)
41‐50	135 (19.8)	432 (63.3)	116 (17)	683 (100)
51‐60	311 (21.7)	882 (61.6)	239 (16.7)	1432 (100)
61 or older	103 (19)	329 (60.6)	111 (20.4)	543 (100)
Education	636 (20.7)	1901 (61.9)	535 (17.4)	3072 (100)
Basic education (primary school)	45 (18.8)	137 (57.3)	57 (23.8)	239 (100)
Matriculation examination	32 (23.2)	81 (58.7)	25 (18.1)	138 (100)
Vocational college	512 (20.6)	1551 (62.3)	428 (17.2)	2491 (100)
University or University of Applied Sciences	46 (23.1)	129 (64.8)	24 (12.1)	199 (100)
Other	1 (20)	3 (60)	1 (20)	5 (100)
Information and communication technology skills	634 (20.6)	1899 (61.8)	538 (17.5)	3071 (100)
Weak	26 (12.2)	109 (51.2)	78 (36.6)	213 (100)
Moderate	344 (20)	1070 (62.3)	304 (17.7)	1718 (100)
Good	264 (23.2)	720 (63.2)	156 (13.7)	1140 (100)
Satisfaction with occupational health care patient portals	633 (21.3)	1871 (63)	466 (15.7)	2970 (100)
Totally unsatisfied	14 (11.9)	55 (46.6)	49 (41.5)	118 (100)
Somewhat unsatisfied	40 (16.1)	177 (71.4)	31 (12.5)	248 (100)
Neither dissatisfied nor satisfied	104 (10.5)	579 (58.2)	312 (31.4)	995 (100)
Somewhat satisfied	380 (27.7)	931 (68)	59 (4.3)	1370 (100)
Totally satisfied	95 (39.7)	129 (54)	15 (6.3)	239 (100)
Guidance needed	632 (21.5)	1860 (63.2)	451 (15.3)	2943 (100)
Totally disagree	273 (31.9)	537 (62.8)	45 (5.3)	855 (100)
Somewhat disagree	112 (24.9)	314 (69.8)	24 (5.3)	450 (100)
Neither disagree nor agree	150 (14.7)	608 (59.7)	260 (25.5)	1018 (100)
Somewhat agree	68 (16.9)	276 (68.7)	58 (14.4)	402 (100)
Totally agree	29 (13.3)	125 (57.3)	64 (29.4)	218 (100)
Fear of unauthorized persons seeing the data	630 (21.3)	1872 (63.2)	458 (15.5)	2960 (100)
Totally disagree	170 (31.2)	344 (63.1)	31 (5.7)	545 (100)
Somewhat disagree	147 (23.9)	422 (68.7)	45 (7.3)	614 (100)
Neither disagree nor agree	151 (15.2)	571 (57.6)	270 (27.2)	992 (100)
Somewhat agree	121 (21.5)	382 (67.7)	61 (10.8)	564 (100)
Totally agree	41 (16.7)	153 (62.4)	51 (20.8)	245 (100)

a OHC: Occupational Health Care.

### The Usability of OHC Patient Portals

**Figure 1.**
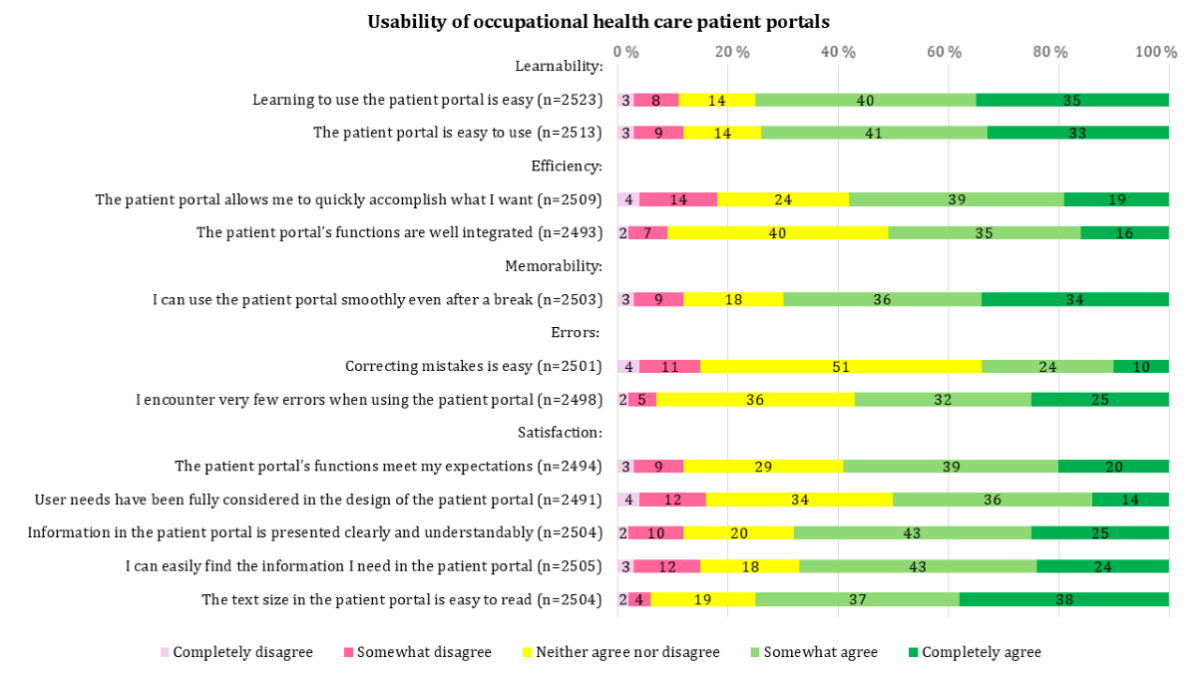
Respondents’ attitude statements regarding the perceived usability of occupational health care patient portals.

According to the results, respondents most positively evaluated aspects related to learnability and ease of use. A large majority (1895/2523, 75.1%) felt that learning to use the patient portal was easy, and most (1861/2513, 74%) experienced the portal as easy to use. Additionally, many respondents (1875/2504, 75%) considered the text size in the portal easy to read. In contrast, the highest levels of disagreement were related to efficiency and user-centered design. Nearly 1 in 5 (451/2509, 18%) respondents disagreed that the portal allows them to quickly accomplish what they want, and 16% (399/2491) disagreed that user needs have been fully considered in the design of the portal. Figure 1 presents respondents’ attitudes toward the usability of OHC patient portals, displayed as relative percentages (without decimals).

The analysis of the GLM assessed the impact of various factors on the usability of OHC patient portals. Satisfaction had the strongest association with the perceived usability of OHC patient portals. Need for guidance and frequency of use were also significantly associated, though to a lesser extent. Fear of unauthorized persons seeing the data, perceived ICT skills, and age were statistically significant but had small effects. The full results are presented in Table 2.

**Table 2.** Results of general linear model analysis on perceived usability of occupational health care patient portals: crude and adjusted models.

	Crude model					Adjusted model				
Variable	Patients, n	Mean (SD)	*F* test (df)	*P* value	η²	Patients, n	Mean (SD)	*F* test (df)	*P* value	η²
Fear of unauthorized persons seeing the data	2492	—	33.3 (4)	<.001	0.051	2427	—	4.49 (4)	.001	0.007
Totally disagree	513	4.0 (0.90)				499	4.0 (0.89)			
Somewhat disagree	565	3.8 (0.73)				558	3.8 (0.73)			
Neutral	720	3.5 (0.73)				694	3.5 (0.73)			
Somewhat agree	501	3.6 (0.70)				489	3.6 (0.70)			
Totally agree	193	3.7 (1.0)				187	3.7 (1.0)			
Need for guidance	2482	—	107.4 (4)	<.001	0.148	2427	—	52.2 (4)	<.001	0.08
Totally disagree	808	4.1 (0.77)				789	4.1 (0.77)			
Somewhat disagree	425	3.8 (0.69)				418	3.8 (0.68)			
Neutral	754	3.4 (0.70)				734	3.4 (0.69)			
Somewhat agree	343	3.4 (0.68)				338	3.4 (0.68)			
Totally agree	152	3.4 (1.1)				148	3.4 (1.1)			
Usage frequency^a^ ^b^	2519	—	110.5 (1)	<.001	0.042	2427	—	34.3 (1)	<.001	0.014
Regularly	635	4.0 (0.77)								
Occasionally	1884	3.6 (0.84)								
Satisfaction^a^ ^b^	2391	—	730.9 (1)	<.001	0.227	2427	—	577.1 (1)	<.001	0.193
Dissatisfied	965	3.2 (0.77)								
Satisfied	1526	4.0 (0.70)								
Perceived ICT^c^ skills^a^ ^b^	2507	—	41.3 (1)	<.001	0.016	2427	—	12.2 (1)	<.001	0.005
Weak	133	3.2 (0.93)								
At least moderate	2374	3.7 (0.82)								
Education	2519	—	2.77 (3)	.04	0.003	—	—	—	—	—
Basic education degree (primary school)	180	3.6 (0.89)								
Matriculation examination	112	3.8 (0.79)								
Vocational college	2052	3.7 (0.77)								
University or University of Applied Sciences	175	3.7 (0.77)								
Gender	2501	—	0.95 (1)	.33	0	—	—	—	—	—
Women	2138	3.7 (0.84)								
Other	363	3.6 (0.82)								
Age (years)^a^ ^b^	2502	—	35.8 (1)	<.001	0.014	2427	—	10.8 (1)	<.001	0.004
50 or younger	896	3.8 (0.80)								
51 or older	1606	3.6 (0.84)								

a As a continuous covariate in the adjusted model; group means not reported.

b As a dichotomic variable in the crude model.

c ICT: information and communication technology.

### The Usefulness of OHC’s Patient Portals

**Figure 2.**
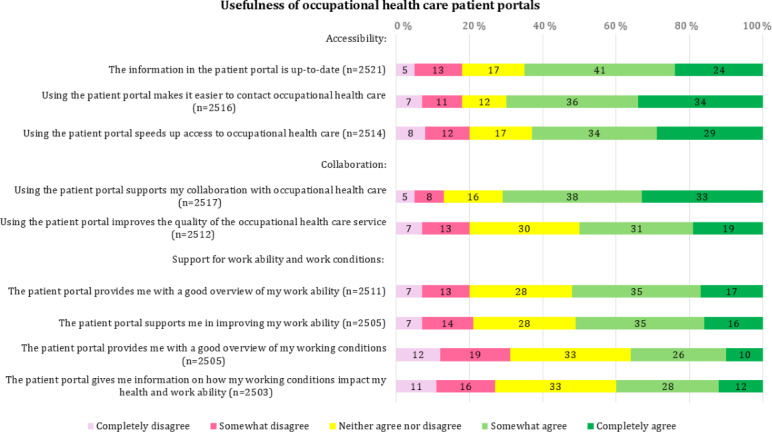
Respondents’ attitude statements regarding the perceived usefulness of occupational health care patient portals.

According to the results, the most positively received aspects of the OHC patient portal were its ability to facilitate easier contact with OHC and to support collaboration. A total of 70.1% (1765/2516) of respondents agreed that using the portal makes it easier to contact OHC, and 70.5% (1774/2517) believed that the portal supports their collaboration with OHC. In contrast, only 52.4% (1316/2511) of respondents felt that the portal provides a good overview of their work ability, and just 51.5% (1291/2505) felt that it supports them in improving it. Regarding working conditions, only 35.5% (891/2505) of respondents felt that the portal offers a good overview of their working conditions, and 39.9% (1000/2503) agreed that it provides information about how those conditions impact their health and work ability. Figure 2 presents respondents’ attitudes toward the usefulness of OHC patient portals, displayed as relative percentages (without decimals).

According to the GLM analysis, satisfaction had the strongest association with the perceived usefulness of OHC patient portals. Frequency of use was also significantly associated with perceived usefulness, though to a lesser extent. The full results are presented in Table 3.

**Table 3.** Results of general linear model analysis on perceived usefulness of occupational health care patient portals: crude and adjusted models.

	Crude model					Adjusted model				
Variable	Patients, n	Mean (SD)	*F* test (df)	*P* value	η²	Patients, n	Mean (SD)	*F* test (df)	*P* value	η²
Fear of unauthorized persons seeing the data	2490	—	5.49 (4)	<.001	0.009	2451	—	0.41 (4)	.80	0.001
Totally disagree	513	3.3 (0.96)				503	3.3 (0.95)			
Somewhat disagree	564	3.2 (0.76)				563	3.2 (0.76)			
Neutral	720	3.1 (0.77)				704	3.1 (0.76)			
Somewhat agree	500	3.2 (0.84)				489	3.2 (0.84)			
Totally agree	193	3.3 (1.1)				192	3.3 (1.0)			
Need for guidance	2480	—	6.58 (4)	<.001	0.011	2451	—	1.52 (4)	.20	0.002
Totally disagree	808	3.3 (0.89)				798	3.3 (0.89)			
Somewhat disagree	425	3.2 (0.77)				422	3.2 (0.78)			
Neutral	752	3.1 (0.80)				741	3.1 (0.80)			
Somewhat agree	343	3.2 (0.82)				340	3.2 (0.82)			
Totally agree	152	3.3 (1.1)				150	3.3 (1.1)			
Usage frequency^a^ ^b^	2523	—	48.08 (1)	<.001	0.019	2451	—	9.80 (1)	.002	0.004
Regularly	634	3.4 (0.87)								
Occasionally	1889	3.1 (0.86)								
Satisfaction^a^ ^b^	2491	—	603.3 (1)	<.001	0.195	2451	—	548.0 (1)	<.001	0.183
Dissatisfied	965	2.7 (0.78)								
Satisfied	1526	3.5 (0.78)								
Perceived ICT^c^ skills^a^	2511	—	2.64 (1)	.10	0.001					
Weak	134	3.1 (0.97)								
At least moderate	2377	3.2 (0.87)								
Education	2523	—	1.41 (3)	.24	0.002					
Basic education (primary school)	181	3.3 (0.98)								
Matriculation examination	112	3.3 (0.81)								
Vocational college	2055	3.2 (0.87)								
University or University of Applied Sciences	175	3.1 (0.80)								
Gender	2505	—	0.001 (1)	.98	0					
Women	2143	3.2 (0.87)								
Other	362	3.2 (0.86)								
Age^a^ (years)	2506	—	2.70 (1)	.1	0.001					
50 or younger	896	3.2 (0.84)								
51 or older	1610	3.6 (0.84)								

a As a continuous covariate in the adjusted model; group means not reported.

b As a dichotomic variable in the crude model.

c ICT: information and communication technology.

## Discussion

### Principal Findings

This study assessed the usability and usefulness of OHC patient portals using a survey. The analysis focused on respondents who reported using the portal either regularly or occasionally, excluding nonusers. Thus, the results reflect the experiences of actual OHC portal users.

Usability was generally rated positively: respondents highlighted ease of use, readability, and ease of learning as the most favorable aspects. Lower ratings were given to task completion speed and the extent to which user needs were considered in the portal’s design. Age and ICT skills were statistically significant factors influencing usability ratings, with respondents aged 50 years or younger and those with strong ICT skills providing more positive assessments.

Usefulness received more mixed feedback: slightly more than half of the respondents felt the portal provided a good overview of their work ability, and just over a third considered it to offer a comprehensive view of their working conditions. Many respondents indicated that the portal did not sufficiently support them in improving their work ability or provide clear information on how working conditions might affect their health and work capacity. In contrast to usability, neither age nor ICT skills significantly influenced how useful the portal was perceived to be.

Additionally, user satisfaction with the OHC patient portal was strongly associated with both its perceived usability and usefulness, with regular users giving it significantly higher ratings than occasional users.

### Comparison With Previous Studies

The findings of this study are consistent with previous research that has identified younger users and those with stronger digital skills as perceiving OHC digital services to be more user-friendly and intuitive [33]. Contrary to earlier studies that highlighted the significance of ICT skills, age, and gender in shaping perceptions of usefulness in digital health services [6,34,35], our study found that these factors did not significantly influence how users perceived the usefulness of digital health services. Moreover, the other studies suggest that both older [36] and younger users [37] generally find OHC patient portals useful, particularly for their clear overview of medical history and treatment plans. The results also showed that satisfied portal users valued its usability more, and those who used the portal regularly perceived its usability as better than occasional users. These results align with previous studies on user satisfaction [3,6,38].

Privacy concerns are a common issue with patient portals in Finland [12,13]. Despite these concerns, patients typically do not restrict the use or disclosure of their information [2]. Interestingly, users of OHC patient portals experience fewer privacy concerns (809/2960, 27.3%) compared to users of the national My Kanta portal (1265/3731, 33.9%) [12]. During the pandemic, privacy concerns among My Kanta users increased even more, with up to half of the respondents expressing concern (1923/3823, 50.3%) [13]. This topic warrants further examination, as patient portals are intended to serve as an essential tool for information exchange and communication between health care professionals and patients.

In this study, only slightly over half of the respondents felt that the portal gave a good overview of their work ability, while only a third found it provided a comprehensive view of their working conditions. Patients generally value access to personal health information [3], and when work-related data—such as the connection between diagnoses and occupational factors—is visible in the portal, it can increase awareness of occupational illnesses [39] and help patients understand how their work environment affects their health and work ability [40]. The patient data displayed in OHC patient portals originates from professionals’ entries in the medical records. Previous studies have found that work-related patient data is rarely documented, even when these issues are discussed with the patient [39,41,42].

### Implications in Occupational Health

In OHC, work ability refers to an individual’s capacity to perform their job, influenced by health, functional capacity, and working conditions [43,44]. OHC visits often focus on evaluating these aspects [45]. To enable OHC patient portals to provide access to this information, the documentation practices must be changed [46,47], particularly toward a more systematic recording of work-related patient data in medical records.

The observed lack of information on work ability and working conditions contrasts with the general expectation that digital services would improve access to information and increase users’ awareness of their own situation [5,6]. When patients have access to information about the hazard, risk, and workload factors of their work and work environments, they can better understand the impact of working conditions on their well-being. This information could enable patients to make informed decisions to provide better opportunities to improve work ability, facilitate discussions with supervisors and OHC professionals on needs for work modifications, and promote the implementation of preventive measures.

To enhance the usability of OHC patient portals, it is important to ensure that the information they contain is comprehensive and easily understandable for patients, and that the portal’s functions are useful to them. For example, the portal could remind users of upcoming periodic occupational health examinations or other job-related health tests, such as spirometry for those exposed to respiratory hazards in the workplace. Additionally, it could allow users to fill out symptom diaries to investigate symptom variation between work and nonwork periods, as well as exposure diaries to monitor the accumulation of exposure [48].

Work, working conditions, and work ability are central areas of OHC [30]. Additionally, OHC patient portals are widely available to many working citizens [28]. Future research should explore how these digital services can be developed to enhance collaboration between patients and OHC professionals.

### Strengths and Limitations

This was a cross-sectional study that examined OHC patients’ experiences with the usability and usefulness of patient portals, aiming to identify factors influencing these aspects and address existing research gaps. The theoretical framework of this study provided a solid foundation for designing the questionnaire and studying the usefulness and ease of use of OHC portals. Although the questionnaire used in this study was self-developed, its reliability is supported by the good Cronbach α values of the composite variables measuring usability and usefulness. This indicates that the questionnaire measured these concepts consistently and strengthens the reliability of the results. Thus, the research methods used in this study were well-suited and provided answers to the RQs while generating new information in line with the research objectives. However, this study has both strengths and limitations.

The strength of this study lies in its uniqueness. To our knowledge, no previous study has systematically assessed patient perceptions of the usability and usefulness of OHC patient portals. Another strength is the large sample size, which ensures a substantial number of observations for analysis and makes the data highly representative. This feature increases confidence in the accuracy and generalizability of the results. The third strength is its timeliness, considering the rapid growth of digital health services. Additionally, calculating the effect size was useful in this study as it provided an objective measure of the magnitude of observed differences. Effect size enabled comparisons between different variables and helped identify clinically significant associations.

The first limitation of this study is the dominant participation of 1 gender; thus, the sample is heavily skewed toward women (2606/3060, 85.2%). This gender imbalance may influence the results and limit the applicability of the findings to a broader population. Future research should aim for a more balanced gender representation to enhance generalizability.

Second, the sample likely includes individuals with higher digital literacy, as the survey was conducted online. This may result in an overrepresentation of participants who are comfortable with digital technologies, potentially excluding those with limited ICT skills. Consequently, the findings may not fully capture the experiences and perspectives of individuals with lower digital literacy.

The third limitation is that this study relies on self-reported data collection. However, certain valuable information can only be obtained through surveys, as was the case in this study. The fourth limitation is the lack of available information about the respondents. As participation in this study was anonymous, we do not know the characteristics of those who declined to participate or did not return the questionnaire. Moreover, despite the statistically significant results obtained in this study, caution should be exercised in interpreting the findings due to the low explanatory power of some results.

### Conclusions

The ability of OHC patient portals to provide information on the work ability and the healthiness and safety of working conditions needs to be improved. In particular, more comprehensive documentation in medical records related to work-related hazards, risks, and workload factors is needed. This could strengthen the role of portals in promoting work ability and in preventing work-related illnesses and accidents, while also increasing patients’ awareness of factors affecting work well-being. Furthermore, improving patient satisfaction with the usability and usefulness of OHC patient portals may lead to increased adoption of portals and, consequently, more equitable access to digital OHC services.

## Abbreviations

GLM: general linear model
ICT: information and communication technology
OHC: occupational health care
RQ: research question

## References

[1] Osborn CY, Mayberry LS, Wallston KA, Johnson KB, Elasy TA (2013). Understanding patient portal use: implications for medication management. J Med Internet Res.

[2] Sääskilahti M, Ojanen A, Ahonen R, Timonen J (2021). Benefits, problems, and potential improvements in a nationwide patient portal: cross-sectional survey of pharmacy customers’ experiences. J Med Internet Res.

[3] Valkonen P, Kujala S, Hörhammer I, Savolainen K, Helminen R, Vartia I (2023). Health self-management of older employees: identifying critical peak experiences of a patient portal. Finn J eHealth eWelfare.

[4] Scheckel B, Schmidt K, Stock S, Redaèlli M (2023). Patient portals as facilitators of engagement in patients with diabetes and chronic heart disease: scoping review of usage and usability. J Med Internet Res.

[5] Eriksson-Backa K, Hirvonen N, Enwald H, Huvila I (2021). Enablers for and barriers to using My Kanta - a focus group study of older adults’ perceptions of the National Electronic Health Record in Finland. Inform Health Soc Care.

[6] Simola S, Hörhammer I, Xu Y (2023). Patients’ experiences of a national patient portal and its usability: cross-sectional survey study. J Med Internet Res.

[7] Kujala S, Simola S, Wang B (2024). Benchmarking usability of patient portals in Estonia, Finland, Norway, and Sweden. Int J Med Inform.

[8] Kujala S, Hörhammer I, Väyrynen A (2022). Patients’ experiences of web-based access to electronic health records in Finland: cross-sectional survey. J Med Internet Res.

[9] Hägglund M, Scandurra I (2022). Usability of the Swedish accessible electronic health record: qualitative survey study. JMIR Hum Factors.

[10] Akyirem S, Wagner J, Chen HN (2024). Recommendations to address barriers to patient portal use among persons with diabetes seeking care at community health centers: interview study with patients and health care providers. JMIR Diabetes.

[11] Vanderhout S, Taneja S, Kalia K, Wodchis WP, Tang T (2025). Patient experiences and perspectives when MyChart is introduced in a large community hospital: mixed methods study. J Med Internet Res.

[12] Kyytsönen M, Vehko T, Jylhä V, Kinnunen UM (2024). Privacy concerns among the users of a national patient portal: a cross-sectional population survey study. Int J Med Inform.

[13] Kainiemi E, Vehko T, Kyytsönen M (2022). The factors associated with nonuse of and dissatisfaction with the national patient portal in Finland in the era of COVID-19: population-based cross-sectional survey. JMIR Med Inform.

[14] Moqbel M, Hewitt B, Nah FFH, McLean RM (2022). Sustaining patient portal continuous use intention and enhancing deep structure usage: cognitive dissonance effects of health professional encouragement and security concerns. Inf Syst Front.

[15] Griffin A, Skinner A, Thornhill J, Weinberger M (2016). Patient portals: who uses them? What features do they use? And do they reduce hospital readmissions?. Appl Clin Inform.

[16] Aljabri D, Dumitrascu A, Burton MC (2018). Patient portal adoption and use by hospitalized cancer patients: a retrospective study of its impact on adverse events, utilization, and patient satisfaction. BMC Med Inform Decis Mak.

[17] Dumitrascu AG, Burton MC, Dawson NL (2018). Patient portal use and hospital outcomes. J Am Med Inform Assoc.

[18] Lear R, Freise L, Kybert M, Darzi A, Neves AL, Mayer EK (2022). Perceptions of quality of care among users of a web-based patient portal: cross-sectional survey analysis. J Med Internet Res.

[19] Huang J, Chen Y, Landis JR, Mahoney KB (2019). Difference between users and nonusers of a patient portal in health behaviors and outcomes: retrospective cohort study. J Med Internet Res.

[20] Kinney AP, Sankaranarayanan B (2021). Effects of patient portal use on patient satisfaction: survey and partial least squares analysis. J Med Internet Res.

[21] Zanaboni P, Kummervold PE, Sørensen T, Johansen MA (2020). Patient use and experience with online access to electronic health records in norway: results from an online survey. J Med Internet Res.

[22] Wang B, Kristiansen E, Fagerlund AJ (2023). Users’ experiences with online access to electronic health records in mental and somatic health care: cross-sectional study. J Med Internet Res.

[23] Freise L, Neves AL, Flott K (2021). Assessment of patients’ ability to review electronic health record information to identify potential errors: cross-sectional web-based survey. JMIR Form Res.

[24] Jormanainen V (2022). Over 89% adoption rate of the nationwide online patient portal in Finland. Stud Health Technol Inform.

[25] Jormanainen V, Lindgren M, Keskimäki I, Kaila M (2023). Use of My Kanta in Finland 2010-2022. Stud Health Technol Inform.

[26] Vehko T, Kaihlanen AM, Kainiemi E, Kyytsönen M, Nadav J, Saukkonen P (2022). Suomalaisten hyvinvointi [Article in Finnish]. https://urn.fi/URN:ISBN:978-952-343-996-2.

[27] Ruotanen R, Kangas M, Tuovinen T, Keränen N, Haverinen J, Reponen J (2021). Finnish e-health services intended for citizens – national and regional development. FinJeHeW.

[28] (2025). Occupational health care statistics of kela 2023 [Article in Finnish]. Official Statistics of Finland.

[29] Nissinen SP, Soini S, Hakulinen H (2021). Kirjatun työkykytiedon tärkeys ja hyödyllisyys työterveyshuollossa – kyselytutkimus työterveyshuollon ammattilaisille [Article in Finnish]. FinJeHeW.

[30] (2021). Translation from Finnish legally binding only in Finnish and Swedish. Occupational Healthcare Act 1383/2001. https://finlex.fi/api/media/statute-foreign-language-translation/212871/mainPdf/main.pdf?timestamp=2001-12-21T00%3A00%3A00.000Z.

[31] Nielsen J (1993). Usability Engineering.

[32] Davis FD (1989). Perceived usefulness, perceived ease of use, and user acceptance of information technology. MIS Q.

[33] Nissinen S, Pesonen S, Toivio P, Sormunen E (2024). Exploring the use, usefulness and ease of use of digital occupational health services: a descriptive correlational study of customer experiences. Digit Health.

[34] Woods SS, Forsberg CW, Schwartz EC (2017). The association of patient factors, digital access, and online behavior on sustained patient portal use: a prospective cohort of enrolled users. J Med Internet Res.

[35] Clarke MA, Fruhling AL, Lyden EL, Tarrell AE, Bernard TL, Windle JR (2021). The role of computer skills in personal health record adoption among patients with heart disease: multidimensional evaluation of users versus nonusers. JMIR Hum Factors.

[36] Huvila I, Rexhepi H, Moll J (2024). Affordance trajectories and the usefulness of online records access among older adults in Sweden. Digit Health.

[37] Hagström J, Blease C, Scandurra I (2024). Adolescents’ reasons for accessing their health records online, perceived usefulness and experienced provider encouragement: a national survey in Sweden. BMJ Paediatr Open.

[38] Yin R, Law K, Neyens D (2021). Examining how internet users trust and access electronic health record patient portals: survey study. JMIR Hum Factors.

[39] Soini S, Ryynänen KR, Nissinen S, Miettunen J, Ala-Mursula L (2025). Assessing the work relatedness of diagnoses in occupational health primary care appointments: a 3-year review of electronic medical records. Occup Environ Med.

[40] Nissinen SP, Soini S, Tarvainen K, Kangas P, Leino T (2021). Työterveyshuollon kirjaamiskäytännöt sairauden liittymisestä työhön ja vaikutuksesta työkykyyn [Article in Finnish]. FinJeHeW.

[41] Weiste E, Vehviläinen S, Leino T, Laitinen J (2018). Ohjauksen vuorovaikutuskäytänteet työterveystarkastuksissa: vertailu ennen koulutusinterventiota ja sen jälkeen [Article in Finnish]. Psykologia.

[42] Lappalainen K, Leino T, Rautio M, Nissinen S (2020). An evaluation of personal health plans in occupational health check-ups. Int J Occup Health Public Health Nurs.

[43] Ilmarinen J (2009). Work ability--a comprehensive concept for occupational health research and prevention. Scand J Work Environ Health.

[44] Tengland PA (2011). The concept of work ability. J Occup Rehabil.

[45] Ikonen A (2012). Primary care visits in the Finnish occupational health services and their connections to prevention and work-related factors. https://helda.helsinki.fi/server/api/core/bitstreams/14435da3-446a-462b-b1a4-9cb662595e89/content.

[46] Meier-Diedrich E, Lyckblad C, Davidge G (2025). Impact of patient online record access on documentation: scoping review. J Med Internet Res.

[47] Kariotis T, Prictor M, Gray K, Chang S (2025). Patient-accessible electronic health records and information practices in mental health care contexts: scoping review. J Med Internet Res.

[48] Fazen LE, Martin BE, Isakari M (2024). Occupational electronic health records: recommendations for the design and implementation of information systems in occupational and environmental medicine practice-ACOEM Guidance Statement. J Occup Environ Med.

